# A new species of the genus *Hexapleomera* Dudich, 1931 from the South Korea, with molecular evidence (Crustacea, Tanaidacea, Tanaididae)

**DOI:** 10.3897/zookeys.739.21580

**Published:** 2018-02-22

**Authors:** Jin Hee Wi, Man-Ki Jeong, Chang-Keun Kang

**Affiliations:** 1 School of Environmental Science & Engineering, Gwangju Institute of Science and Technology, Gwangju 500-712, South Korea; 2 The Fisheries Science Institute, Chonnam National University, Daehak-ro, Yeosu 59626, South Korea

**Keywords:** genetic analysis, *Hexapleomera*, sexual dimorphism, Tanaidacea, Tanaidomorpha

## Abstract

Two populations of new species are described for *Hexapleomera* Dudich, 1931 from the southeastern coast and Jeju island of South Korea (north west Pacific). The specimens were collected using a light trap set overnight at the entrance near a pier or harbour. *Hexapleomera ulsana* **sp. n.** is clearly differentiated from other species in the genus by the uropod with five articles, a maxillule palp with four distal setae, the maxilliped coxa with three proximal setae, the epignath with short and blunt spiniform seta, the propodus of pereopods 2–3 with three ventral setae, and the maxilla with a rugged shape of the distal margin. Differences of mitochondrial cytochrome c oxidase subunit I (mtCOI) gene observed between two populations of *H. ulsana* from different regions (Ulsan and Jeju Island) and between *H. ulsana* and *H. urashima* (Japan) were 1.1 % and 32.4 %, respectively. Two genetically-close populations differed in the setae on pleopod 3, the proximal setae on the maxilliped coxa, and the ventral setae on pereopods 2–3, which showed that geographical distance affected the morphological divergence. In addition, a comprehensive comparison with previous records of *Hexapleomera* was conducted and close examinations on the appendages, known to have morphological variations between the individuals of one species and/or between different genders, were carried out based on new species and discussed herein.

## Introduction

Tanaidaceans are represented by almost 1,200 mostly marine species, and these are distributed from almost any type of marine habitat (Błażewicz-Paszkowycz and Bamber 2012), providing a main food source for fishes (Nagelkerken et al. 2000, Edgar 2008). Tanaidaceans are small benthic crustaceans with a benthic life cycle and low dispersion rates. Some of the taxa are dispersed by marine vertebrates (Moore 1894, Morales-Vela et al. 2008, Bamber et al. 2009), floating algae (Bamber and Costa 2009), anthropogenic transport such as shipping (Bamber et al. 2009), and by prevailing sea-currents (Bamber 1998). The genus *Hexapleomera* Dudich, 1931 has been found to be taxon commensal on turtles, or manatees and regarded to show cosmopolitan distribution (Heard et al. 2004, Edgar 2008, Morales-Vela et al. 2008). Recent studies revealed that some species of the genus inhabit diverse environments, such as on the hulls of yachts and benthic sediments (Bamber et al. 2009, Bamber 2012). Species of *Hexapleomera* in the present study were found in harbour benthic habitats.

The genus *Hexapleomera* was defined by Sieg (1980) with characteristics of the narrow anterior pereonites, five distinct pleonites, the differentiation between the pleopods 1–2 and pleopod 3, and the distal article of the uropod without reduction. Males represent the sexual dimorphism in the antennule, antenna, and cheliped. Up to quite recently, *Hexapleomera robusta* described by Sieg (1980) from the Atlantic coasts of Brazil, or the Galapagos Island, has been considered as a cosmopolitan species (Bamber 2012). In particular, due to the absence of the type material of *H. robusta*, many described species of *Hexapleomera* having a commensal ecology with motile organisms have been regarded as *H. robusta*: specimens recorded by Riggio (1976, 1996), Sieg (1980), Heard et al. (2004), Edgar (2008), Morales-Vela et al. (2008), and Bamber et al. (2009). Recently, *H. edgari* Bamber, 2012 and *H. satella* Bamber, 2012, which have also been known as *H. robusta*, were newly assigned as morphologically distinct species. Currently, *Hexapleomera* consists of nine species including species of this study (WoRMS 2017) and *H. crassa* Riggio, 1975 in status of ‘nomen nudum’. As with most other genera of Tanaididae, some features of the genus *Hexapleomera*, which contains the relative lengths of antennule articles 1 and 2, the number of setae consisting of the setal row represented as lacinia mobilis of the mandible, and the number and shape of uropod articles, are considered to vary with the growth of individuals (Larsen and Wilson 1998, Edgar 2008). In addition, some previous descriptions and figures of the species within the genus were limited to the highly dimorphic male [e.g. *H. edgari* and *H. moverleyi* (Edgar 2008)]. Therefore, a more detailed analysis of the female morphology is necessary for a full understanding of the species (Bamber 2012). Moreover, a combined molecular and morphological analysis can be an adequate solution to a taxonomic problem caused by cryptic speciation and/or invasions of new habitats (Edgar 2008). This paper examined the morphological and genetic characteristics based on mature females and males of species of Korean *Hexapleomera* in different regions to reveal if the geographical distance affected morphological features.

## Materials and methods

The material was obtained from the bottom of entrances near two harbours of South Korea: Ulsan (35°22'7.47"N; 129°35'25.47"E) in January 2016 and Jeju Island (33°14.0'N; 126°22.6'E) in May 2016, using a light trap set. The light trap set was made with a PVC pipe (10 cm in diameter, 50 cm long) and a LED lamp on the mouth, placed on mud-sandy bottom of 3m in depth at the seawalls in harbors after sunset. The samples caught overnight in the light trap were filtered through a plankton net with 350 μm mesh at dawn. The specimens were preserved in 99 % alcohol solution. The individuals were dissected under a dissection microscope (Nikon SMZ745T) in CMC-10 aqueous mounting medium (Masters, Wood Dale, IL, USA), mounted on slides, and then sealed with high-quality nail varnish. Drawings were generated using a differential interference contrast microscope (Nikon Y-IM) that was equipped with a drawing tube. The total body length was measured from the tip of the rostrum to the pleotelson apex in the dorsal view. Scale bars are given in mm. The morphological terminology follows Larsen (2003). The type and other material examined were deposited in the collections of the Marine Biodiversity Institute of Korea (**MABIK**), Seocheon, South Korea.

### Molecular analysis

**DNA extraction, amplification, sequencing, and analysis**

The total genomic DNA was extracted from five specimens of *Hexapleomera* (*H. ulsana*: two females MABIK CR00240699, MABIK CR00240701, and one male MABIK CR00240704; Jeju population of *H. ulsana*: one female MABIK CR00240707, and one male MABIK CR00240710). To extract genomic DNA, a centrifuge tube (1.5 mL) each containing 90 μL of 10 % Chelex suspension (Bio-Rad Laboratories Inc.), 10 μL of Proteinase K (10 mg/ml, iNtRON Biotechnology, Inc.) and grinded tissues were incubated at 56 °C for 3 hours. The extracted genomic DNAs were used as templates to amplify the target regions on the mtCOI gene. Polymerase chain reaction (PCR) was performed on a Mastercycler PCR thermal cycler (Eppendorf Co.). A pair of primer for COI was jgLCO1490 and jgHCO2198 (Geller et al. 2013). PCR mixtures contained 16 μL of deionized water, 1 μL of each primer (10 μM), 2 μL of DNA template and PCR premix (BiONEER Co.). The cycle parameters consisted of initial denaturation at 95 °C for 3 min, denaturation at 95°C for 30 s, annealing at 48 °C (45 s) and extension at 72 °C (1 min) repeated for 40 cycles, with a final extension time of 7 min at 72 °C. The results of the PCR amplification were confirmed on 1.0 % agarose gels by using ethidium bromide staining. The obtained PCR products were purified and sequenced at the Macrogen Inc. facilities (Seoul, Korea). The sequences were aligned with those from *H. urashima* Tanabe et al. (2017) (accession number LC322243–322248) on a Chromas software version 2.33 (Technelysium Pty. Ltd.).

## Systematics

### Order Tanaidacea Dana, 1849

#### Family Tanaididae Nobili, 1906

##### Subfamily Pancolinae Sieg, 1980

###### Tribe Pancolini Sieg, 1980

####### Genus *Hexapleomera* Dudich, 1931

######## Hexapleomera ulsana sp. n.

Taxon classificationAnimaliaTanaidaceaTanaididae

http://zoobank.org/168A8768-3D0A-437D-99C6-028B62310729

**Figure 1.**
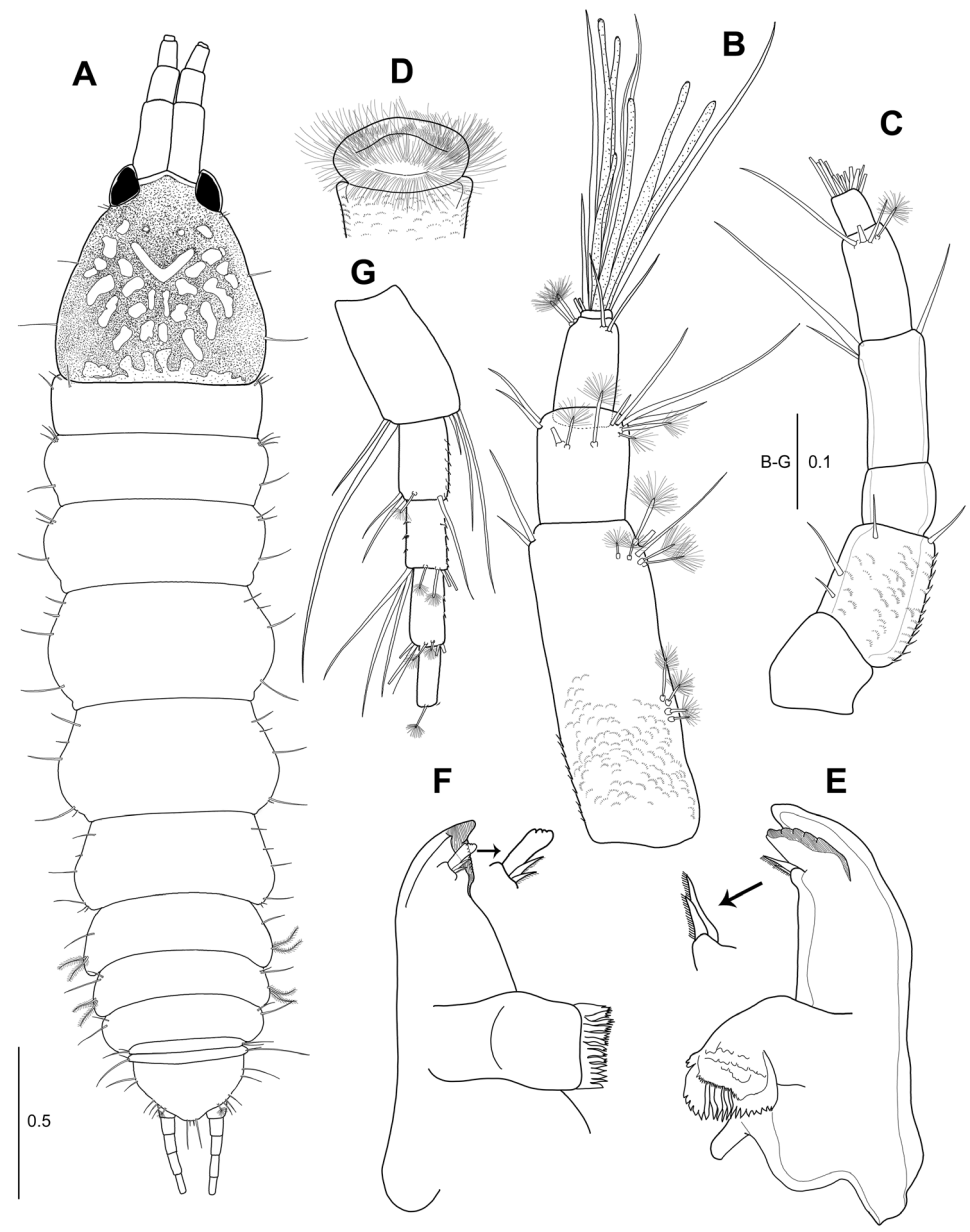
*Hexapleomera ulsana* sp. n., female holotype, **A** habitus, dorsal **B** antennule **C** antenna **D** labrum **E** left mandible **F** right mandible. Scale bars in mm.

**Figure 2.**
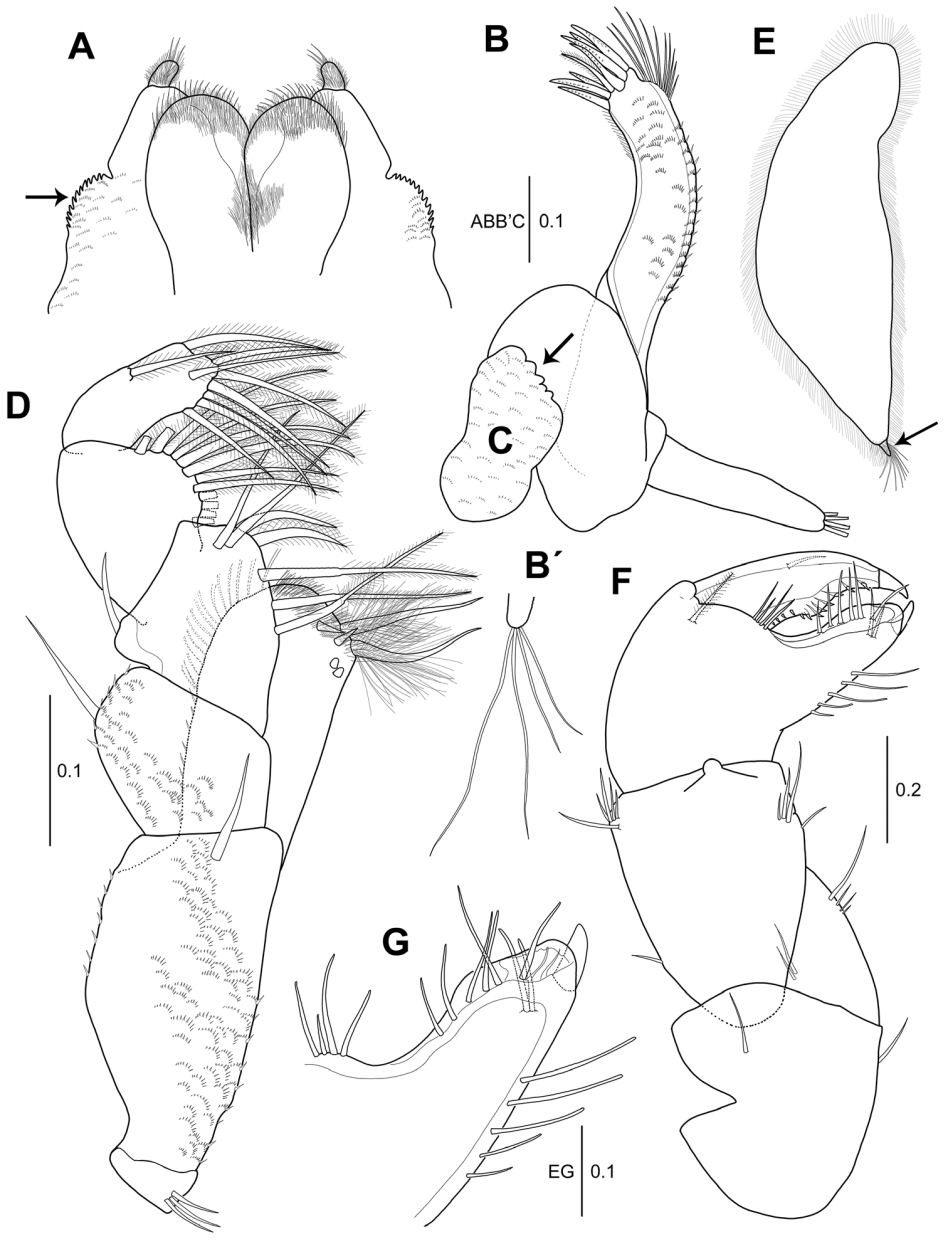
*Hexapleomera ulsana* sp. n., female holotype **A** labium **B** maxillule **B**´ distal setae of maxillule palp **C** maxilla **D** maxilliped **E** epignath **F** cheliped **G** fixed finger of cheliped. Scale bars are given in mm.

**Figure 3.**
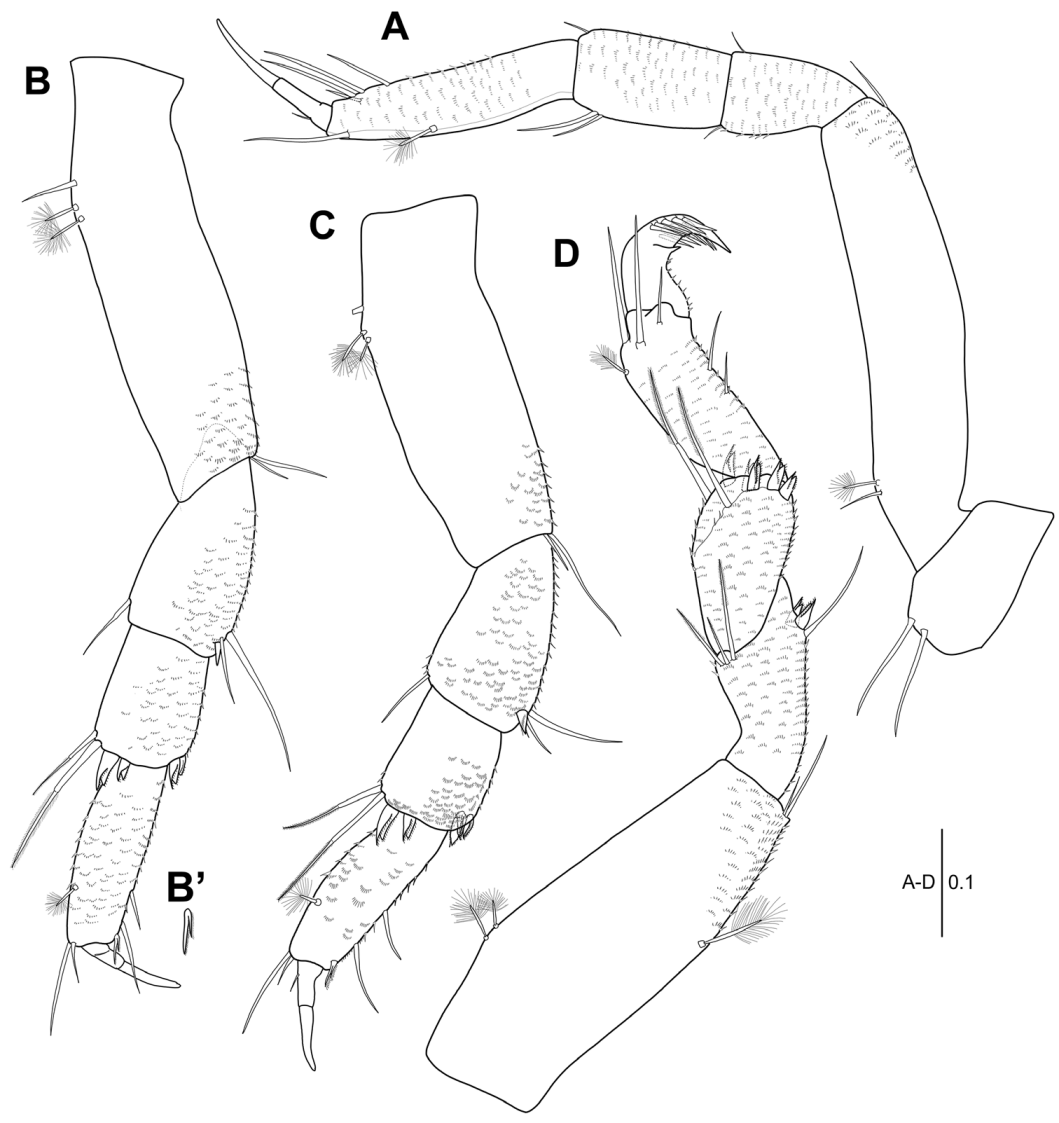
*Hexapleomera ulsana* sp. n., female holotype, **A** pereopod 1 **B** pereopod 2 **C** pereopod 3 **D** pereopod 4. Scale bars in mm.

**Figure 4.**
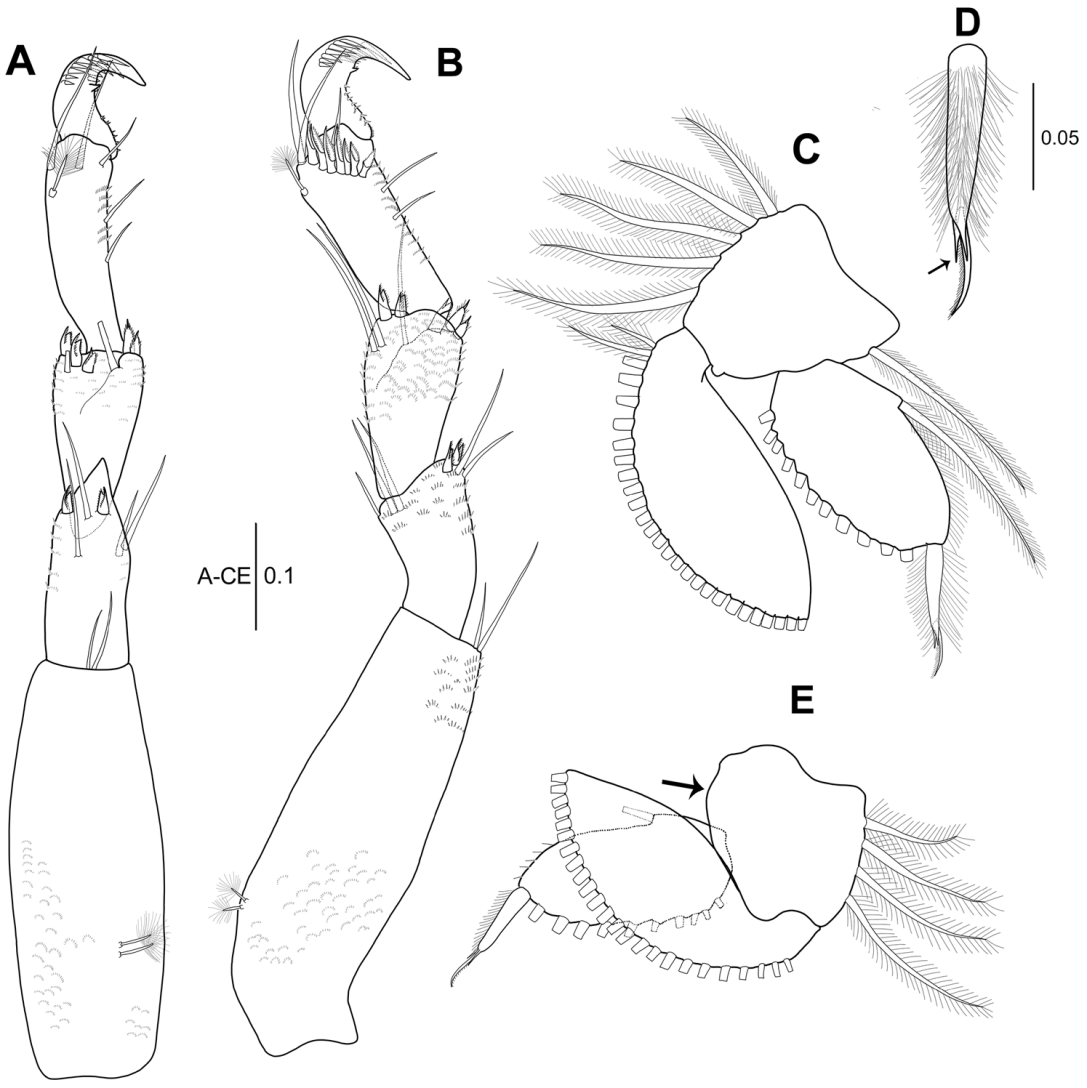
*Hexapleomera ulsana* sp. n., female holotype, **A** pereopod 5 **B** pereopod 6 **C** pleopod 1 **D** distal seta of pleopod1 endopod **E** pleopod 3. Scale bars in mm.

**Figure 5.**
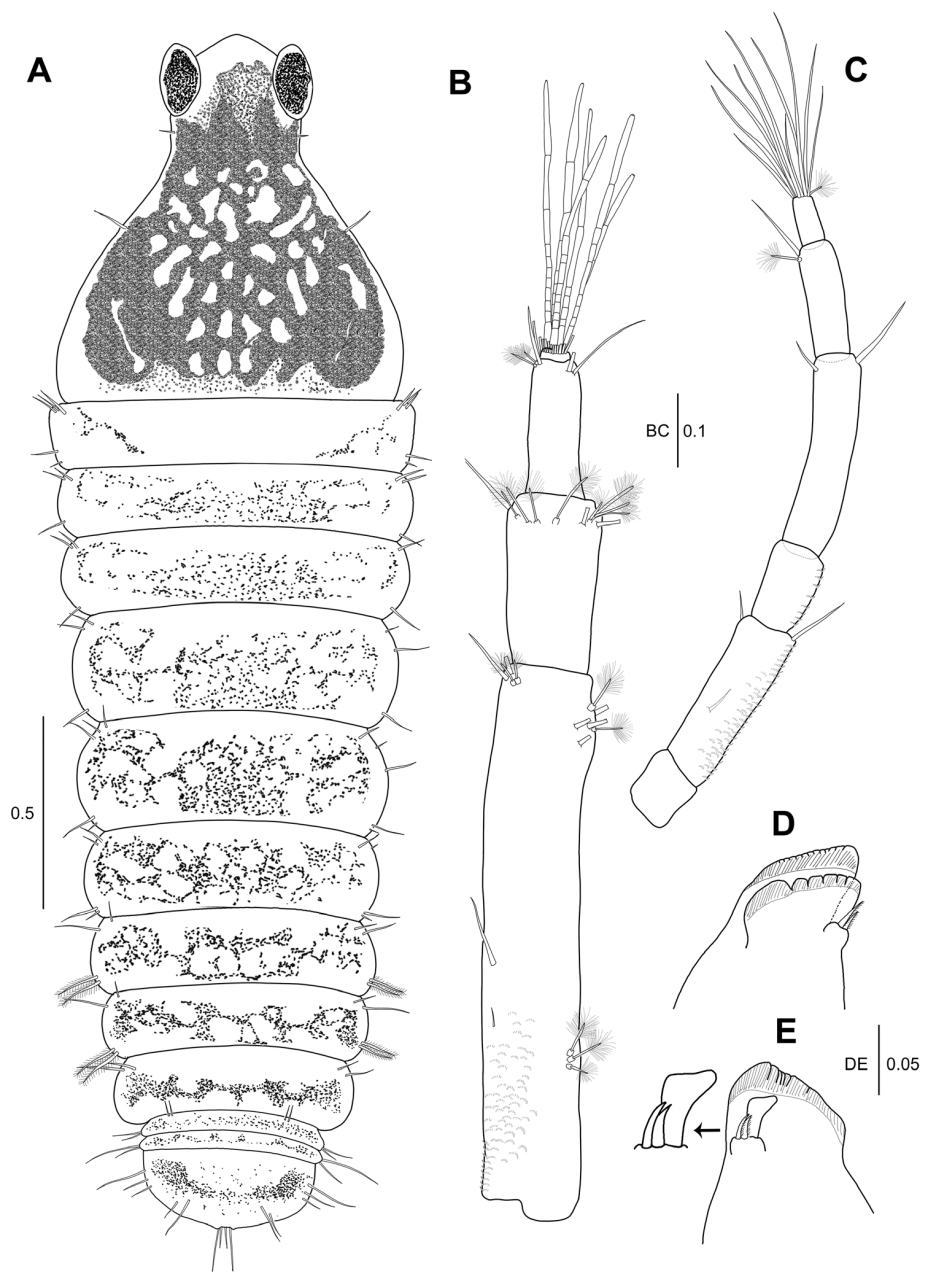
*Hexapleomera ulsana* sp. n., male allotype, **A** habitus, dorsal, all pigmentations except that on carapace not presented **B** antennule **C** antenna **D** left mandible, incisor, lacinia mobilis, and setal row **E** right mandible, incisor, lacinia mobilis, and setal row. Scale bars in mm.

**Figure 6.**
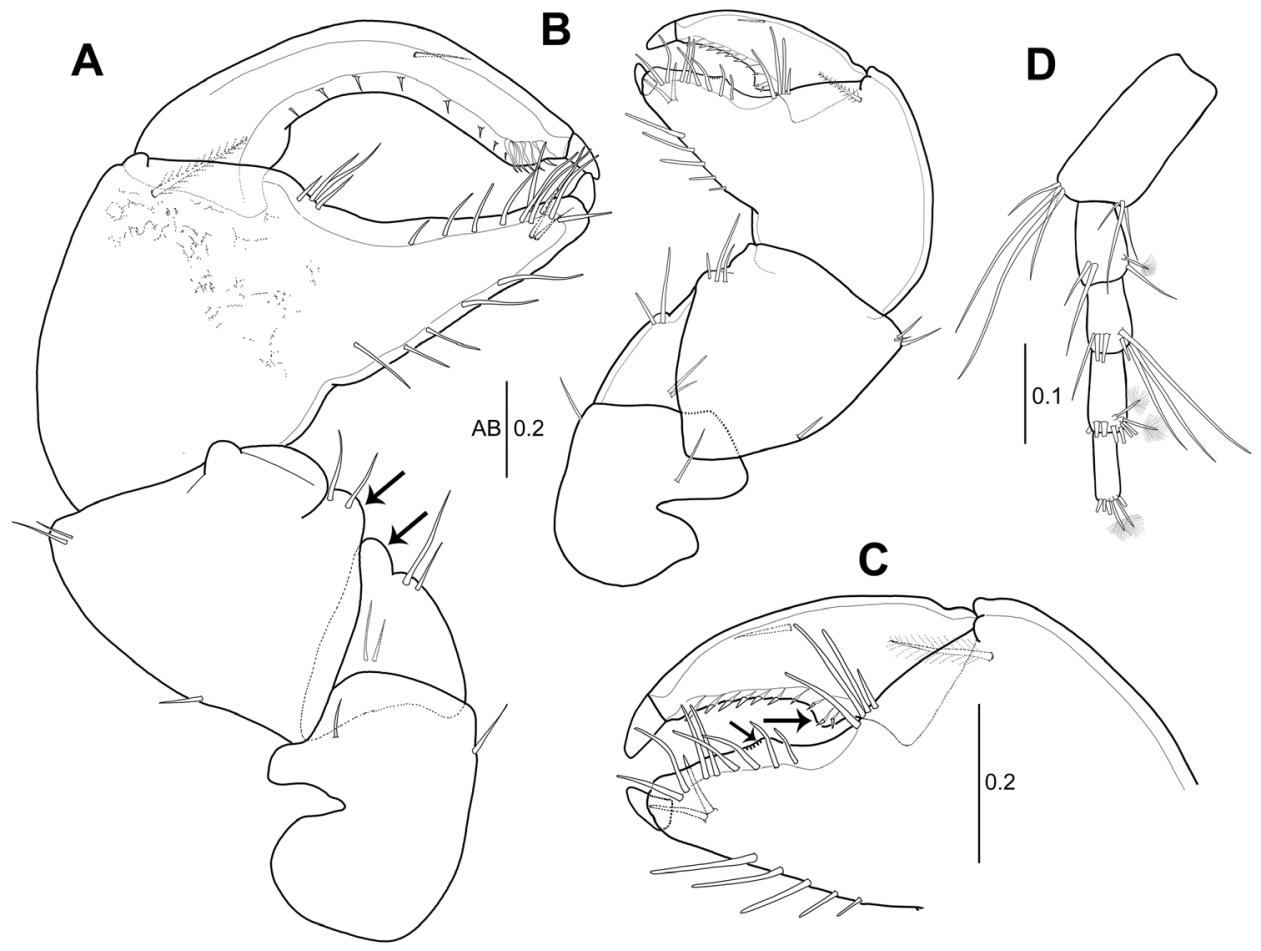
*Hexapleomera ulsana* sp. n., male allotype, **A** cheliped **B–C** male paratype **B** cheliped **C** cheliped chela **D** allotype male, uropod. Scale bars in mm.

Figs 1 , 2 , 3 , 4 , 5 , 6

######### Type-specimens.

**Holotype.** (MABIK CR00235364) ovigerous female dissected and mounted in eight slides, collected from the Ulsan of Korea (35°22'7.47"N, 129°21'25.47"E) in January 2016. **Allotype.** (MABIK CR00235366) male dissected and mounted in four slides, same locality as for holotype. **Paratypes.** Four females partially dissected and mounted in three slides (MABIK CR00235365), two slides (MABIK CR00240699), five slides (MABIK CR00240700), and two slides (MABIK CR00240701); two intact females in one vial (MABIK CR00240702); four males partially dissected and mounted in two slides (MABIK CR00235367), four slides (MABIK CR00240703), one slide (MABIK CR00240704), one slide (MABIK CR00240705); two intact males in one vial (MABIK CR00240706). Same locality as for holotype.

######### Diagnosis.

Uropod endopod 4-articulate. Pleopods 1–3 endopod with one seta on inner margin. Pleopod 3 basal article with four outer setae and no inner seta; pleopods 1–2 each with six outer setae and one inner seta on basal article. Pereopod 1 coxa lacking apophysis and having two dorsal setae. Pereopods 2–3 propodus with two simple setae and one bifurcated pinnate spiniform seta on ventral margin. Mandible setal row consists of two pinnate setae. A maxillule palp with four distal setae. Maxilliped coxa with three proximal setae; endite bearing two long plumose setae, two short plumose spiniform setae, and two simple spiniform setae. Dactylus of male cheliped with spinules along cutting edge.

######### Description of female.

***Body*** (Fig. 1A): With developed brood sacs each with 20 eggs. Length 3.4 mm, 4.2 times as long as wide. Chocolate brown pigmentation on most of dorsal surface, including antennule and cheliped, patterned with un-pigmented lacunae. Cephalothorax with densely coalescing flecks but pereonites, pleonites, and pleotelson with scattered spots.

***Cephalothorax*** (Fig. 1A): Posterolateral margin rounded, anteriorly narrowing, as long as wide, 22 % of body length, with two anterolateral simple setae, one mid-lateral seta, and posterolateral seta.

***Pereon*** (Fig. 1A): About 55 % of body length, 2.3 times as long as wide, with several fine setae along antero, mid, and posterolateral margins. Pereonites 1–6 proportional lengths of 10.6: 13.3: 16.4: 14.9: 22.2: 21.5: 16.0.

***Pleon*** (Fig. 1A): Pleonites 1–5 18.6 % of body length, 1.2 times as long as wide. Pleonites 1–3 gradually decreasing in width. Pleonite 1 0.8 times as long as pereonite 6, 0.3 times as long as wide, with one simple seta and two plumose setae on lateral margin. Pleonite 2 0.7 times as long as pleonite 1, 0.2 times as long as wide, with two simple setae and two plumose setae on lateral margin. Pleonite 3 almost as long as pleonite 2, 0.3 times as long as wide, with two simple setae. Pleonites 4 and 5 equal in length, each with two simple setae on lateral margin. Pleotelson 0.9 times as long as pleonite 1, half as long as wide, with two simple setae on mid-lateral margin, three simple setae and one broom seta on sub-posterolateral margin, and four simple setae of unequal length terminally.

***Antennule*** (Fig. 1B): 4-articulate, proportional lengths of articles 60.5: 21.1: 16.9: 1.5. Length 68 % of cephalothorax. Article 1 2.7 times as long as wide, outer margin with four medial broom setae and five broom setae and two simple setae on distal margin; inner margin with two distal simple setae. Article 2 1.2 times as long as wide, with four broom setae and seven simple setae on distal and subdistal margins. Article 3 1.5 times as long as wide, with two broom setae and two simple setae on distal and subdistal margins. Article 4 very short, with seven simple setae and six aesthetascs.

***Antenna*** (Fig. 1C): 6-articulate, slightly longer than antennule, proportional lengths of articles 16.6: 23.2: 12.3: 21.8: 18.8: 7.3. Article 1 naked. Article 2 with four simple setae, microtrichia, and setules along outer margin. Article 3 naked. Article 4 with three simple setae on distal margin. Article 5 with two simple and two broom setae. Article 6 with twelve simple setae on distal margin.

***Labrum*** (Fig. 1D): Rounded, ornamented with numerous setules and microtrichia.

***Left mandible*** (Fig. 1E): Incisor stout, smooth. Lacinia mobilis wide, with five denticles along distal margin. Setal row with two pinnate spiniform setae. Molar broad, corrugate and with several distal teeth. *Right mandible* (Fig. 1F): Incisor with several small denticles on distal margin. Lacinia mobilis reduced to blunt spine partly fused with incisor with small denticles on distal margin. Setal row with two pinnate setae of unequal length. Molar broad, with several distal teeth.

***Labium*** (Fig. 2A): Wide, all lobes covered with setules on distal margin, with twelve lateral proximal spines (arrowed); labial palp rounded, 1.4 times as long as wide, with numerous setules.

***Maxillule*** (Fig. 2B, B’): Endite with seven strong spiniform setae and one slender setulose seta on distal margin and patch of fine simple setae near base of terminal setae on outer margin, surface ornamented with microtrichia, and palp with four distal setae.

***Maxilla*** (Fig. 2C): With sinuous distal margin (arrowed), covered with microtrichia.

***Maxilliped*** (Fig. 2D): Coxa with three inner proximal simple setae. Basis twice as long as wide, with single subdistal seta, and covered with microtrichia. Palp article 1 with one outer subdistal seta. Palp article 2 with one outer seta and seven inner plumose setae. Palp article 3 with twelve inner plumose setae. Palp article 4 with twelve plumose setae. Endite with setules on distal margin, two long densely plumose setae, two setulose spiniform setae, and two round simple spiniform setae.

***Epignath*** (Fig. 2E): With short blunt plumose seta terminally and fringed by setules (arrowed).

***Cheliped*** (Fig. 2F, G): Basis 1.1 times as long as wide, with single seta each on dorsodistal and ventral margin. Merus triangular, with four ventral setae and two dorsal setae. Carpus 1.1 times as long as basis, 1.4 times as long as wide, with four ventro-subdistal setae and one dorso-subproximal and five dorsodistal setae. Propodus 1.3 times as long as carpus, 1.9 times as long as wide, with one plumose inner seta and four simple setae near insertion of dactylus. Fixed finger with five simple ventral setae, two inner simple setae, and six simple setae along cutting edge. Cutting edge distally expanded into rounded lamella. Dactylus with twelve small denticles along cutting edge and inner medial simple seta.

***Pereopod 1*** (Fig. 3A): Coxa without small apophysis, with two simple setae. Ischio-basis 4.3 times as long as wide, with one broom seta, one ventrodistal and one dorsodistal simple setae on dorsoproximal margin and one ventrodistal simple seta. Merus 0.3 times as long as ischio-basis, with one short ventrodistal simple seta. Carpus 1.2 times as long as merus, with two dorsal simple setae and one ventral simple seta. Propodus 2.1 times as long as carpus, with one distal simple seta and one broom seta on dorsal margin, three simple setae on ventro-subdistal margin, and one inner subdistal seta. Ischio-basis, merus, carpus, and propodus covered by microtrichia. Dactylus and unguis combined 0.6 times as long as propodus. Dactylus with simple seta on dorso-subproximal margin. Unguis 1.3 times as long as dactylus.

***Pereopod 2*** (Fig. 3B, B’): Ischio-basis 3.2 times as long as wide, with two broom setae and one simple seta on dorsoproximal margin and two ventrodistal simple setae. Merus 0.4 times as long as ischio-basis, with two simple setae and one spiniform seta on ventrodistal margin and one dorsodistal simple seta. Carpus 0.6 times as long as merus, with two spiniform setae and two slender setae on dorsodistal margin and two ventrodistal spiniform setae. Propodus 1.5 times as long as carpus, with two distal simple setae of unequal length and one dorsal broom seta and two simple setae and one short bifurcate pinnate spiniform seta on ventral margin (Fig. 3B’). Dactylus and unguis combined half as long as propodus. Ischio-basis, merus, carpus, and propodus covered by microtrichia. Dactylus with one dorsoproximal simple seta. Unguis 1.4 times as long as dactylus.

***Pereopod 3*** (Fig. 3C): Ischio-basis 2.8 times as long as wide, with two broom setae and one simple seta on dorso-subproximal margin and two ventrodistal simple setae. Merus half as long as ischio-basis, setation equal to pereopod 2. Carpus 0.6 times as long as merus, with three ventrodistal setulose spiniform setae and two setulose spiniform setae and two slender setae on dorsodistal margin. Propodus 1.4 times as long as carpus, setation equal to that of pereopod 2. Ischio-basis, merus, carpus, and propodus covered by microtrichia. Dactylus and unguis combined as long as that of pereopod 2. Unguis 1.5 times as long as dactylus.

***Pereopod 4*** (Fig. 3D): Ischio-basis 2.7 times as long as wide, with two dorso-subproximal broom setae, one ventro-subdistal broom seta, and two ventrodistal simple setae. Merus 0.6 times as long as ischio-basis, with three dorsodistal setae and two setulose spiniform setae and one seta on ventro-subdistal margin. Carpus 0.7 times as long as merus, with two slender setae and five setulose spiniform setae on distal margin. Propodus 1.2 times as long as carpus, with one dorso-subdistal broom seta, three subdistal simple setae, and two ventral slender simple setae. Ischio-basis, merus, carpus, and propodus covered by microtrichia. Dactylus and unguis fused into claw, with comb-like lateral rows of five flattened setae distally.

***Pereopod 5*** (Fig. 4A): Ischio-basis 2.5 times as long as wide, with two dorso-subproximal broom setae and two ventrodistal simple setae. Merus 0.4 times as long as ischio-basis, with four simple setae and two seutlose spiniform setae. Carpus 0.8 times as long as merus, with five setulose spiniform plus two simple setae on distal margin. Propodus 1.4 times as long as carpus, setation equal to that of pereopod 4. Ischio-basis, merus, carpus, and propodus covered by microtrichia. Dactylus and unguis similar to those of pereopod 4.

***Pereopod 6*** (Fig. 4B): Ischio-basis 3.1 times as long as wide, with two dorso-subproximal broom setae and two ventrodistal setae. Merus 0.4 times as long as ischio-basis, with three dorsodistal setae and two setulose spiniform setae and two simple setae on ventrodistal margin. Carpus slightly longer than merus, with five setulose spiniform setae and three setae. Propodus 1.1 times as long as carpus, with seven flattened denticulate spiniform setae and one simple seta on subdistal margin, one dorsal broom seta, two distal setae, and two ventral slender simple setae. Ischio-basis, merus, carpus, and propodus covered by microtrichia. Dactylus and unguis similar to those of pereopod 5.

***Pleopod 1*** (Fig. 4C, D): Basal article as long as wide, with one inner and five outer plumose setae. Exopod with 27 plumose setae along outer margin. Endopod with one inner and eleven outer plumose setae and one robust spiniform plumose seta bearing two spines and one setulose seta (arrowed in Fig. 4D).

***Pleopod 2*** (not figured): Similar to pleopod 1, except for outer margin of exopod with smaller plumose setae (26).

***Pleopod 3*** (Fig. 4E): Basal article lacking inner seta (arrowed) and with four outer plumose setae. Exopod with 24 outer plumose setae. Endopod with one inner plumose seta and ten outer plumose plus one robust setae.

***Uropod*** (Fig. 1A, G): Basal article 1.7 times as long as wide, with three outer distal setae and two inner distal setae. Endopod 4-articulate.

######### Description of male.

*Body* (Fig. 5A): Length 3.4 mm, three times as long as wide. Pigmentation similar to female. Cephalothorax pear-shaped, one-third of body length, 1.1 times as long as wide, with antero- and mid-lateral simple setae, tapering towards to rostrum.

***Pereon*** (Fig. 5A): 44 % of body length, 1.3 times as long as wide, with several fine setae along lateral margins. Pereonites 1–6 proportional lengths of 13.7: 13.2: 15.6: 21.0: 20.5: 16.0.

***Pleon*** (Fig. 6A): Pleonites 1–5 18.1 % of body length, 0.7 times as long as wide. Pleonites 1–3 gradually decreasing in width. Pleonite 1 almost equal length of pereonite 6, 0.2 times as long as wide, with two simple and two plumose setae on lateral margin. Pleonite 2 0.7 times as long as pleonite1, 0.2 times as long as wide, with two simple and two plumose setae on lateral margin. Pleonite 3 slightly shorter than pleonite 2, 0.2 times as long as wide, with two simple setae on anterolateral margin. Pleonites 4 and 5 combined half as long as pleonite 3, 0.7 times as wide as pleonite 3, each with two lateral simple setae. Pleonite 4 with two anterodorsal setae. Pleotelson (Fig. 5A) about 0.7 times as long as pereonite 1, 0.3 times as long as wide, with three simple setae on sub-anterolateral margin, one broom seta and two simple setae on sub-posterolateral margin, and four simple setae of unequal length on distal margin.

Appendages similar to those of female except for antennule, antenna, mandible, maxilliped, cheliped, and uropod:

***Antennule*** (Fig. 5B): 4-articulate, elongate. Proportional lengths of articles 63.3: 20: 15.4: 1.3. Length 1.7 times as long as cephalothorax. Article 1 5.6 times as long as wide, outer margin with three subproximal broom setae and two broom plus four simple setae on distal margin; inner margin with two medial simple setae and two broom and two simple setae on distal margin. Article 2 1.8 times as long as wide, with three simple setae and eight broom setae on subdistal margin. Article 3 2.3 times as long as wide, with two broom setae and four simple setae on distal margin. Article 4 small, with six aesthetascs and several simple setae on distal margin.

***Antenna*** (Fig. 5C): 6-articulate, 0.8 times as long as antennule. Proportional lengths of articles 9.3: 24.1: 12.5: 29.5: 17.2: 7.4. Article 1 naked. Article 2 with one medial simple seta and two distal simple setae. Article 3 ornamented with microtrichia. Article 4 longest, with two distal simple setae. Article 5 with one broom seta and one simple seta on inner distal margin. Article 5 shortest, with one broom seta and ten simple setae on distal margin.

***Left mandible*** (Fig. 5D): Incisor with small denticles along distal margin. *Right mandible* (Fig. 6E): Lacinia mobilis without distal denticles.

***Cheliped*** (Fig. 6A): Much stouter than that of female. Basis 1.3 times as long as wide, with one ventrodistal simple seta and one dorsodistal simple seta. Merus subtriangular, ventrodistally protruded (arrowed), with two ventral simple setae and two dorsal setae. Carpus 1.1 times as long as basis, almost as long as wide, with two ventral protrusions (arrowed), two ventral simple setae, one dorsoproximal simple seta, and two dorsodistal simple setae. Propodus 2.1 times as long as carpus, 1.9 times as long as wide, with one inner plumose seta and four simple setae near dactylus insertion. Fixed finger with five ventral simple setae, two inner distal setae, and ten simple setae along cutting edge. Dactylus with 14 small denticles along cutting edge and inner medial seta.

***Uropod*** (Fig. 6D): Basal article 2.2 times as long as wide, with four outer distal simple setae and three inner distal simple setae.

***Cheliped of smaller male*** (Body length 2.1 mm) (Fig. 6B, C): Basis as long as wide. Merus with one ventral protrusion, two ventral simple setae, and two dorsal simple setae. Carpus with weakly developed protrusion on ventral margin. Propodus 1.5 times as long as carpus, 1.9 times as long as wide, with one inner plumose seta and four simple setae near dactylus insertion. Fixed finger with five ventral simple setae, two inner distal setae, small protrusion bearing small denticles (arrowed in Fig. 6C), and eight simple setae along cutting edge. Dactylus not extended and just weakly curved, and with proximal protrusion (arrowed in Fig. 6C) and small denticles along cutting edge.

######### Etymology.

The specific name refers to Ulsan, a harbour city near the type locality.

######### Remarks.

*Hexapleomera ulsana* sp. n. can be differentiated from other species of the genus by the following combination of characteristics (Table 1): in both sexes, 1) the uropod has five articles; 2) the basal article of the pleopod 3 lacks inner seta and has four outer setae; 3) the pereopod 1 coxa lacks apophysis; 4) the maxillule palp has four distal setae; in the maxilliped, 5) a coxa has three distal setae; 6) the basis has one subdistal seta; 7) each setal row of the right and left mandibles has two pinnate setae; and 8) the propodus of pereopods 2–3 each has two slender simple setae and one bifurcate spiniform seta on the ventral margin. Just as *H. urashima*, *Hexapleomera ulsana* lacks an inner seta on the pleopod basal article, has the same number of distal setae on the maxillule endite (6), setae of the mandible setal row (2), and ventral setae on the pereopods 2–3 propodus (3), but can be distinguished by the pereopod 1 coxa lacking apophysis (vs. presence), the number of uropod article (5 vs. 4), and the number of proximal seta on the maxilliped coxa (3 vs. 2). In addition, the findings of the maxilla forming a rugged shape on the distal margin and an epignath with substantially short and blunt spiniform distal seta are noted. In other species of *Hexapleomera*, the maxilla represents a simple and round shape, and the distal seta of the epignath is much slender. The cheliped of the smaller male is very similar to those of females without any trace of oostegites; however, it differs in the location of a protrusion on the dactylus and fixed finger. Particularly, the cutting edge of the fixed finger of the female is smooth, without a protrusion, while it has a weak protrusion with small denticles in the smaller male.

**Table 1.** Morphological comparison between nine species within the genus *Hexapleomera*.

Key characters \ species	*H. bultidactyla*	*H. edgari*	*H. moverleyi*	*H. robusta* *sensu* Moore	*H. robusta* *sensu* Sieg	*H. satella*	*H. ulsana*	*H. urashima*	*H. wombat*
	F/M	F/M	F/M	F/M	F/M	F/M	F/M	F/M	F/M
**Number of uropod articles**	4/4	4/4	–/5	4/–	4/4	4/4	5/5	4/4	5/5
**Number of inner seta on pleopod 3 basal article**	0/0	1/1	–/1	–	0/0	0/0	0/0	0/0	0/0
**Pereopod 1 coxa with apophysis**	yes	yes	yes	–	no	yes	no	yes	no
**Number of seta on maxillule palp**	4	5	5	8	8	4	4	6	5
**Maxilliped**									
Basis setae	2	2	1	–	2	2	1	1~2	1
Coxa setae	2	3	2	–	2	2	3	2	3
Endite with distal plumose setae	yes	yes	yes	–	yes	no	yes	yes	no
**Right/Left Mandibles**									
Setae number of setal row	1	1	1	–	2	1	2	2	1
**Pereopods 2–3**									
Ventral setae on propodus	2, 2	4, 4	2, 2	–	3, 2	1, 2	3, 3	3, 3	2, 3
**Habitat**	aquaculture fouling	turtles	epifaunal	turtles	turtles	benthic/epifaunal	benthic/epifaunal	turtles	yacht hulls

### Jeju population of *Hexapleomera ulsana*

Figs 7–12

#### Material examined

Yerae (33°27'30.0"N, 126°20'18.0"E), Jeju Island of Korea in May 2016. Ovigerous female dissected and mounted in five slides (MABIK CR00235368). Male dissected and mounted in five slides (MABIK CR00235370). Three females partly dissected and mounted in five slides (MABIK CR00235369), one slide (MABIK CR00240707), three slides (MABIK CR00240708); two partly dissected females in one vial (MABIK CR00240709); three males dissected and mounted in one slide (MABIK CR00235371), one slide (MABIK CR00240710), one slide (MABIK CR00240711); two partly dissected males in one vial (MABIK CR00240712).

**Figure 7.**
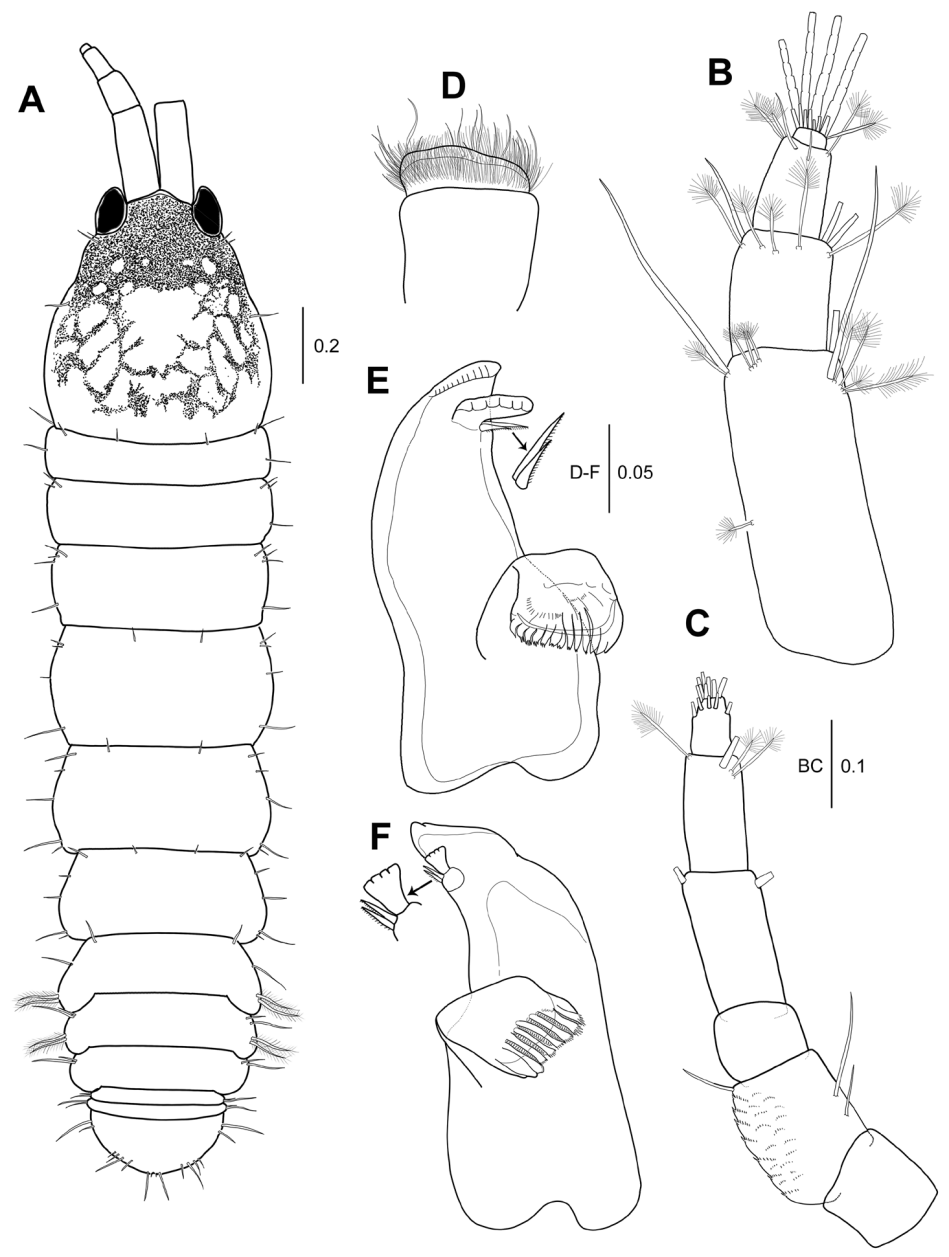
*Hexapleomera ulsana* sp. n., Jeju population, female, **A** habitus, dorsal, all pigmentations except that on carapace not presented **B** antennule **C** antenna **D** labrum **E** left mandible **F** right mandible. Scale bars in mm.

**Description of female** (with a budding of oostegites). *Body* (Fig. 7A): With developed brood sacs each with 18 eggs. Length 2.6 mm, 4.3 times as long as wide. Chocolate brown pigmentation on most of dorsal surface, including antennule and cheliped, patterned with un-pigmented lacunae, cephalothorax with densely coalescing flecks focused on anterior part and eyes. Cephalothorax 1.1 times as long as wide, 26 % of body length, with two pairs of simple slender setae on antero- and mid-lateral margins.

***Pereon*** (Fig. 7A): About 51 % of body length, 2.2 times as long as wide, with several fine setae along lateral margins. Pereonites 1–6 proportional lengths of 10.8: 12.5: 16.5: 22.6: 21.1: 16.5.

***Pleon*** (Fig. 7A): Pleonites 1–5 18% of body length. Pleonite 1 0.9 times as long as pereonite 6, 0.35 times as long as wide, with one simple seta on anterodorsal margin, simple seta on sub-anterolateral margin, and two plumose setae on posterolateral margin. Pleonite 2 0.8 times as long as pleonite 1, 0.3 times as long as wide, with two simple setae on sub-anterolateral margin and two plumose setae on sub-posterolateral margin. Pleonite 3 0.8 times as long as pleonite 2, 0.2 times as long as wide, with two simple setae on sub-anterolateral margin. Pleonites 4 and 5 each with two simple setae on lateral margin. Pleotelson (Fig. 7A): 0.7 times as long as pleonite 1, 0.4 times as long as wide, with two simple setae of unequal length on sub-anterolateral margin, three simple setae and one broom seta on sub-posterolateral margin, and four simple setae of unequal length on posterior margin.

***Antennule*** (Fig. 7B): 4-articulate. Proportional lengths of articles 57.1: 22.4: 17.3: 3.2. Length 0.7 times as long as cephalothorax. Article 1 2.5 times as long as wide, with one medial broom seta and distally eight broom setae and three simple setae. Article 2 1.2 times as long as wide, with five broom setae and three simple setae on distal margin. Article 3 1.5 times as long as wide, with five broom setae and two simple setae. Article 4 with seven simple setae, one broom seta, and four aesthetascs.

***Antenna*** (Fig. 7C): 6-articulate, 1.4 times as long as antennule. Proportional lengths of articles 15.4: 22.4: 11.4: 22.8: 18.5: 9.5. Article 1 naked. Article 2 with three simple setae and microtrichia. Article 3 naked. Article 4 with two distal simple setae. Article 5 with one simple seta and three broom setae on distal margin. Article 6 with twelve simple setae on distal and sub-distal margins.

***Labrum*** (Fig. 7D): With numerous setules.

***Left mandible*** (Fig. 7E): Incisor with small denticles along distal margin. Lacinia mobilis wide, with six denticles along distal margin. Setal row with two pinnate spiniform setae of unequal length. Molar similar to that of Ulsan population. *Right mandible* (Fig. 7F): Incisor distally smooth. Lacinia mobilis reduced to blunt spine with four distal denticles. Setal row with two pinnate spiniform setae of similar length. Molar similar to that of left mandible.

**Figure 8.**
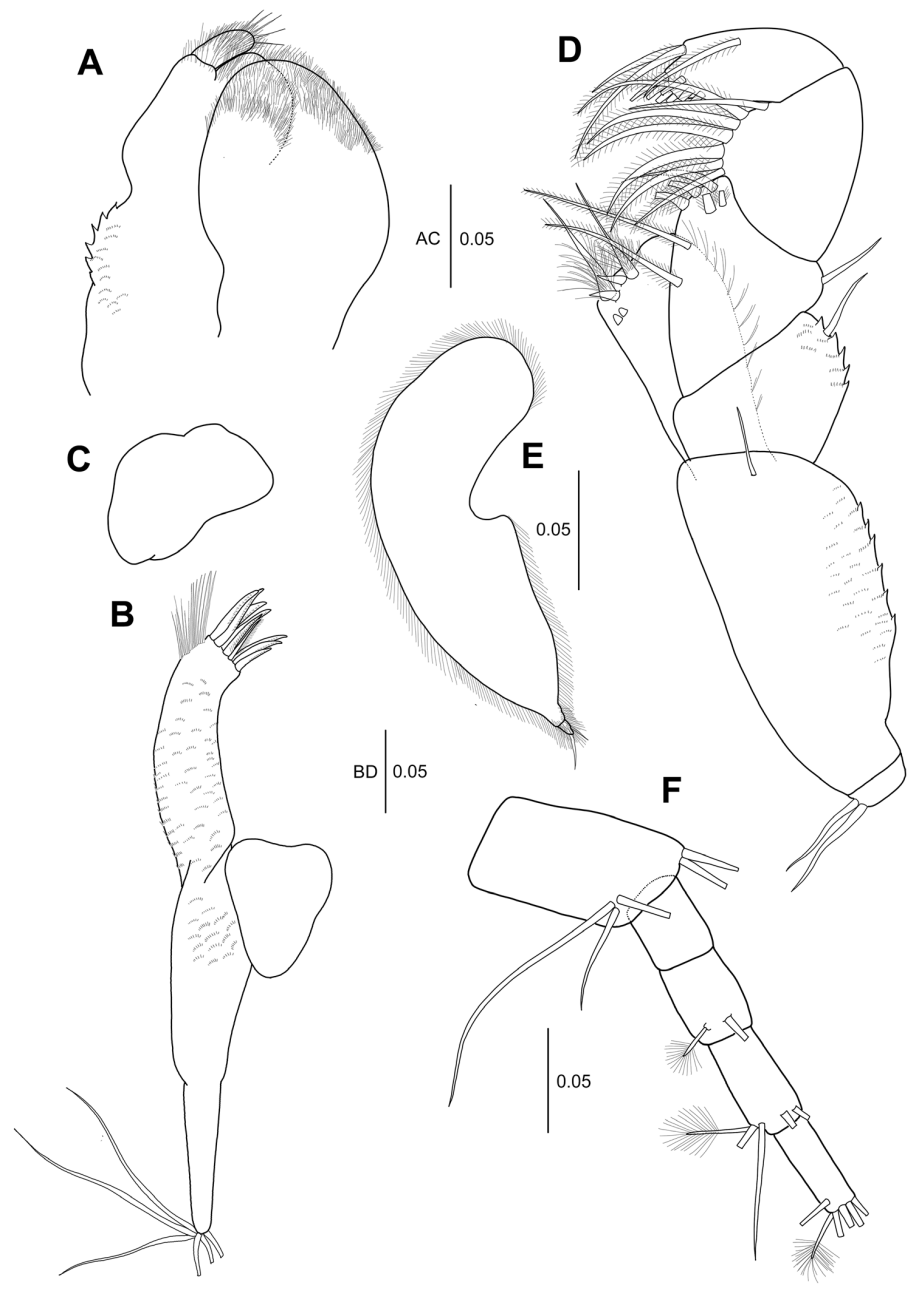
*Hexapleomera ulsana* sp. n., Jeju population, female, **A** labium **B** maxillule **C** maxilla **D** maxilliped **E** epignath **F** uropod. Scale bars in mm.

***Labium*** (Fig. 8A): With six lateral spines; labial palp 1.9 times as long as wide, covered with setules.

***Maxillule*** (Fig. 8B): Endite with seven strong setulate spiniform setae and one slender setulose seta on distal margin and patch of simple slender setae on outer subdistal margin; palp with six distal setae.

***Maxilla*** (Fig. 8C): Distal margin simple.

***Maxilliped*** (Fig. 8D): Coxa with two inner proximal simple setae. Basis 2.2 times as long as wide, with one distal seta. Palp article 1 with one outer subdistal seta. Palp article 2 with one outer seta and seven inner plumose setae. Palp article 3 with seven inner plumose setae. Palp article 4 with ten plumose setae. Endite with two plumose setae, two short simple spiniform setae, and two blunt spiniform setae on distal and subdistal margins. *Epignath* (Fig. 8E): Similar to that of Ulsan population.

**Figure 9.**
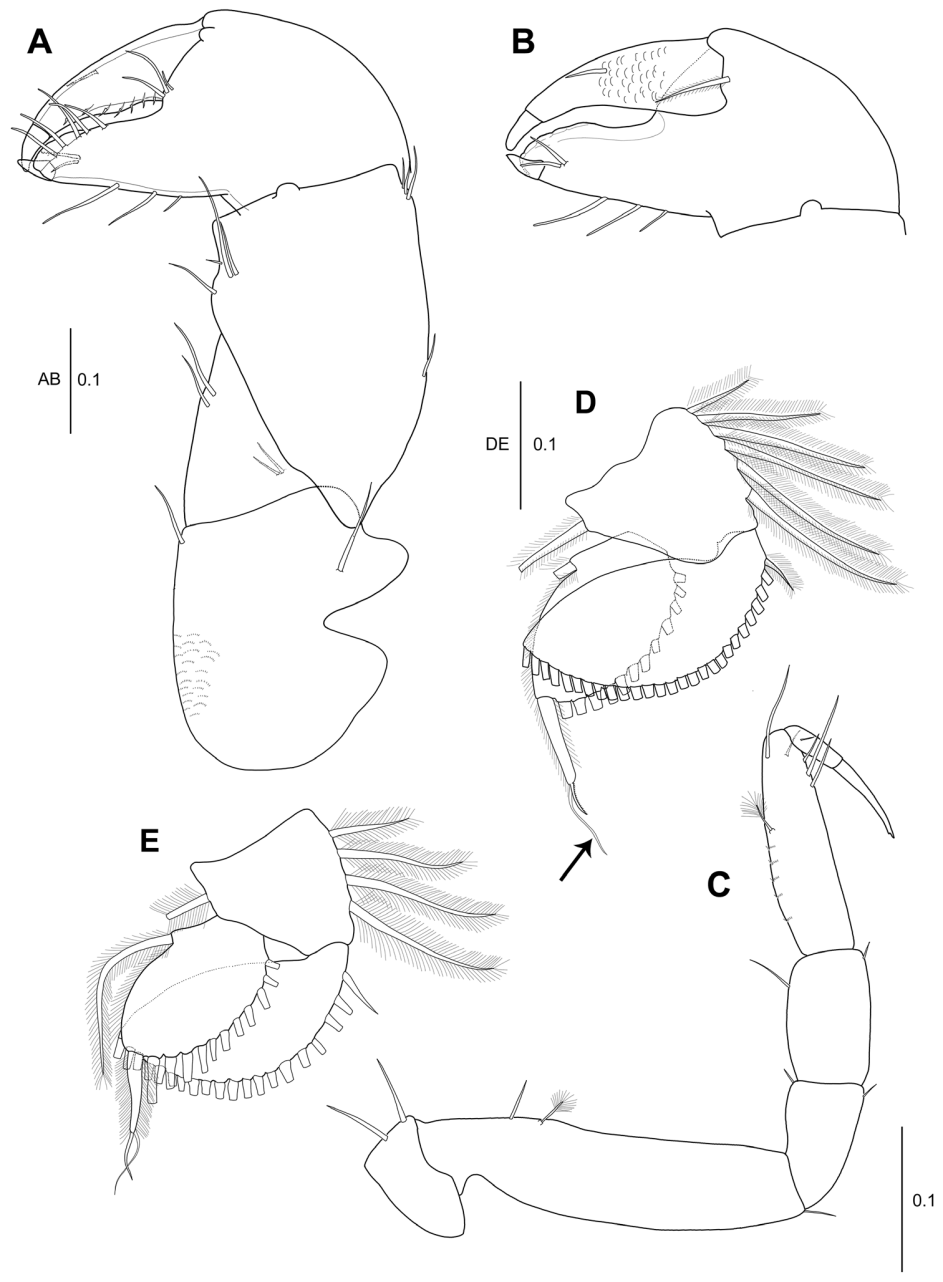
*Hexapleomera ulsana* sp. n., Jeju population, female, **A** cheliped **B** cheliped chela, inner surface **C** pereopod 1 **D** pleopod 1 **E** pleopod 3. Scale bars in mm.

***Cheliped*** (Fig. 9A, B): Basis 1.2 times as long as wide, with one dorsal seta, one ventral seta, and microtrichia on ventroproximal margin. Merus with two ventral setae and two dorsal setae. Carpus 1.2 times as long as basis, 1.6 times as long as wide, with four ventral setae, three dorsodistal setae, and one dorsomedial seta. Propodus 1.1 times as long as carpus, 1.9 times as long as wide, with three simple setae and one inner setulose seta near insertion of dactylus (Fig. 9B). Fixed finger with three simple setae on ventral margin, two inner simple setae, and five outer simple setae along cutting edge. Dactylus with seven small denticles along cutting edge and inner surface with one simple seta medially and covered with squama-like ornamentations.

***Pereopod 1*** (Fig. 9C): Coxa with two simple setae, without apophysis. Ischio-basis 4.2 times as long as wide, with one simple seta and one broom seta on dorso-subproximal margin and one simple ventrodistal seta. Merus 0.4 times as long as ischio-basis, with one ventrodistal seta and one dorsodistal seta. Carpus as long as merus, with one ventrodistal seta and one dorsodistal seta. Propodus 1.7 times as long as carpus, with three ventro-subdistal setae, one dorsomedial broom seta, one inner subdistal seta, and one dorso-subdistal seta. Dactylus and unguis combined 0.7 times as long as propodus. Dactylus with simple seta on dorsoproximal margin. Unguis 1.5 times as long as dactylus.

**Figure 10.**
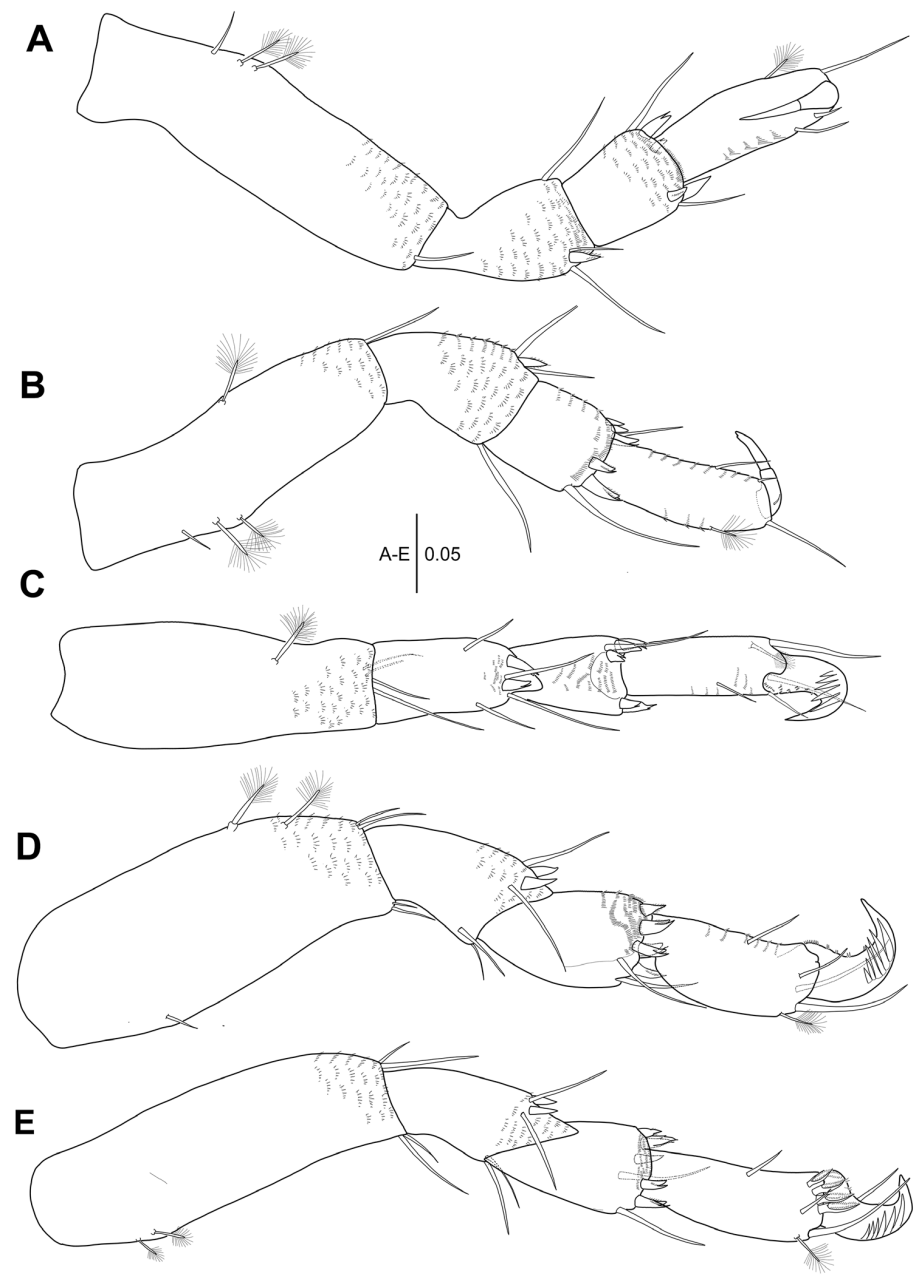
*Hexapleomera ulsana* sp. n., Jeju population, female, **A** pereopod 2 **B** pereopod 3 **C** pereopod 4 **D** pereopod 5 **E** pereopod 6. Scale bars in mm.

***Pereopod 2*** (Fig. 10A): Ischio-basis 3.3 times as long as wide, with two broom setae and one simple seta on dorsal margin and one ventrodistal simple seta. Merus 0.4 times as long as ischio-basis, with one setulose spiniform seta and two simple setae on ventrodistal margin and one dorsodistal simple seta. Carpus 0.7 times as long as merus, with four setulose spiniform setae and two simple setae on distal margin. Propodus 1.6 times as long as carpus, with two simple ventral setae of unequal length, one simple dorsodistal seta, and one dorso-subdistal broom seta. Dactylus and unguis combined 0.6 times as long as propodus. Dactylus with short proximal simple seta. Unguis 1.6 times as long as dactylus.

***Pereopod 3*** (Fig. 10B): Ischio-basis 3.1 times as long as wide, with two broom setae and one simple seta on dorsal margin, one ventromedial broom seta, and one ventrodistal simple seta. Merus half as long as ischio-basis, setation equal to that of pereopod 2. Carpus 0.6 times as long as merus, with three setulose spiniform setae and one simple seta on ventrodistal margin and two setulose spiniform setae and one simple seta on dorsodistal margin. Propodus 1.8 times as long as carpus, setation equal to that of pereopod 2. Dactylus and unguis combined half as long as propodus. Dactylus with subproximal simple seta. Unguis 1.3 times as long as dactylus.

***Pereopod 4*** (Fig. 10C): Ischio-basis 2.5 times as long as wide, with one broom seta and two ventrodistal simple setae. Merus half as long as ischio-basis, with two setulose spiniform setae and two ventrodistal and two dorsodistal simple setae. Carpus 0.7 times as long as merus, with five setulose spiniform setae and two simple setae. Propodus 1.4 times as long as carpus, with one ventral and one mid-inner distal simple setae, two dorsodistal setae, and one dorsal broom seta. Dactylus and unguis fused into claw, with comb-like lateral rows of five flattened setae.

***Pereopod 5*** (Fig. 10D): More robust than in pereopod 4. Ischio-basis with two ventral broom setae, one dorsal simple seta, two ventrodistal simple setae, and two dorsodistal simple setae. Carpus 0.8 times as long as merus. Setation of merus, carpus, and propodus equal to those of pereopod 4. Propodus 1.2 times as long as carpus. Claw with comb-like lateral rows of six flattened setae.

***Pereopod 6*** (Fig. 10D): Ischio-basis three times as long as wide, with two dorso-subproximal broom setae and two ventrodistal simple setae. Merus half as long as ischio-basis, setation equal to that of pereopod 5. Carpus equal to that of pereopod 5. Propodus with four flattened denticulate setae on distal margin, one ventral simple and one mid-inner setae, two dorsodistal setae, and one dorso-subdistal broom seta. Claw similar to that of pereopod 5.

***Pleopod 1*** (Fig. 9D): Basal article almost as long as wide, with one inner plumose seta and six outer plumose setae. Exopod with 27 outer plumose setae. Endopod with one inner and twelve outer plumose setae and one robust spiniform seta bearing one setulose spine and one long seta (arrowed).

***Pleopod 2*** (not figured): Basal article and exopod similar to those of pleopod 1. Endopod with one inner and eleven outer plumose setae and one robust seta.

***Pleopod 3*** (Fig. 9E): Basal article with four outer plumose setae and one inner plumose seta. Exopod with 20 outer plumose setae. Endopod with one inner and eleven outer plumose setae and one robust seta.

***Uropod*** (Fig. 8F): Basal article 1.7 times as long as wide, with two inner distal setae and three outer distal setae. Endopod with four articles.

**Figure 11.**
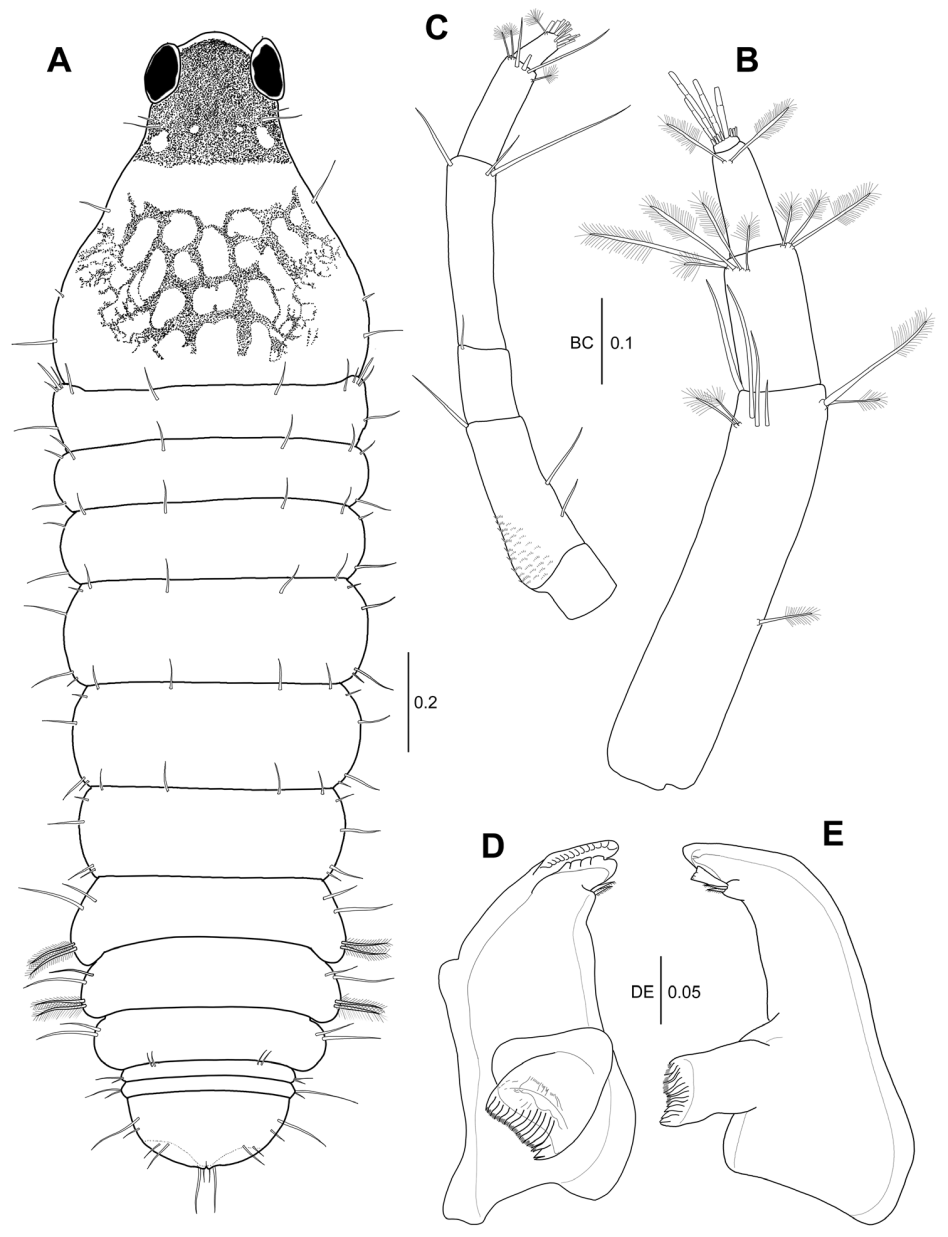
*Hexapleomera ulsana* sp. n., Jeju population, male, **A** habitus, dorsal **B** antennule **C** antenna **D** left mandible **E** right mandible. Scale bars in mm.

**Description of male.** *Body* (Fig. 11A): Length 2.4 mm, 3.6 times as long as wide. Pigmentation similar to female. Cephalothorax 31 % of body length, 1.1 times as long as wide, with two simple setae on sub-anterolateral margin, one mid-lateral seta, and two sub-posterolateral setae.

***Pereon*** (Fig. 11A): 43 % of body length, 1.6 times as long as wide. Pereonites 1–6 with several simple setae along lateral margin, proportional lengths of 10.6: 12.7: 16.2: 20.8: 21.7: 18.0.

***Pleon*** (Fig. 11A): Pleonites 1–5 20.3 % of body length, 0.8 times as long as wide. Pleonite 1 0.8 times as long as pereonite 6, 0.3 times as long as wide, with two simple setae and two plumose setae on lateral margin. Pleonites 2 and 3 each slightly shorter than pleonite 1, with two simple setae and two plumose setae. Pleonites 4 and 5 each with two lateral simple setae of unequal length. Pleonite 4 with two pairs of two simple setae on dorsoproximal margin. Pleotelson 0.8 times as long as pleonite 1, half as long as wide, with two simple anterior setae, two medial simple setae, and four distal simple setae.

**Figure 12.**
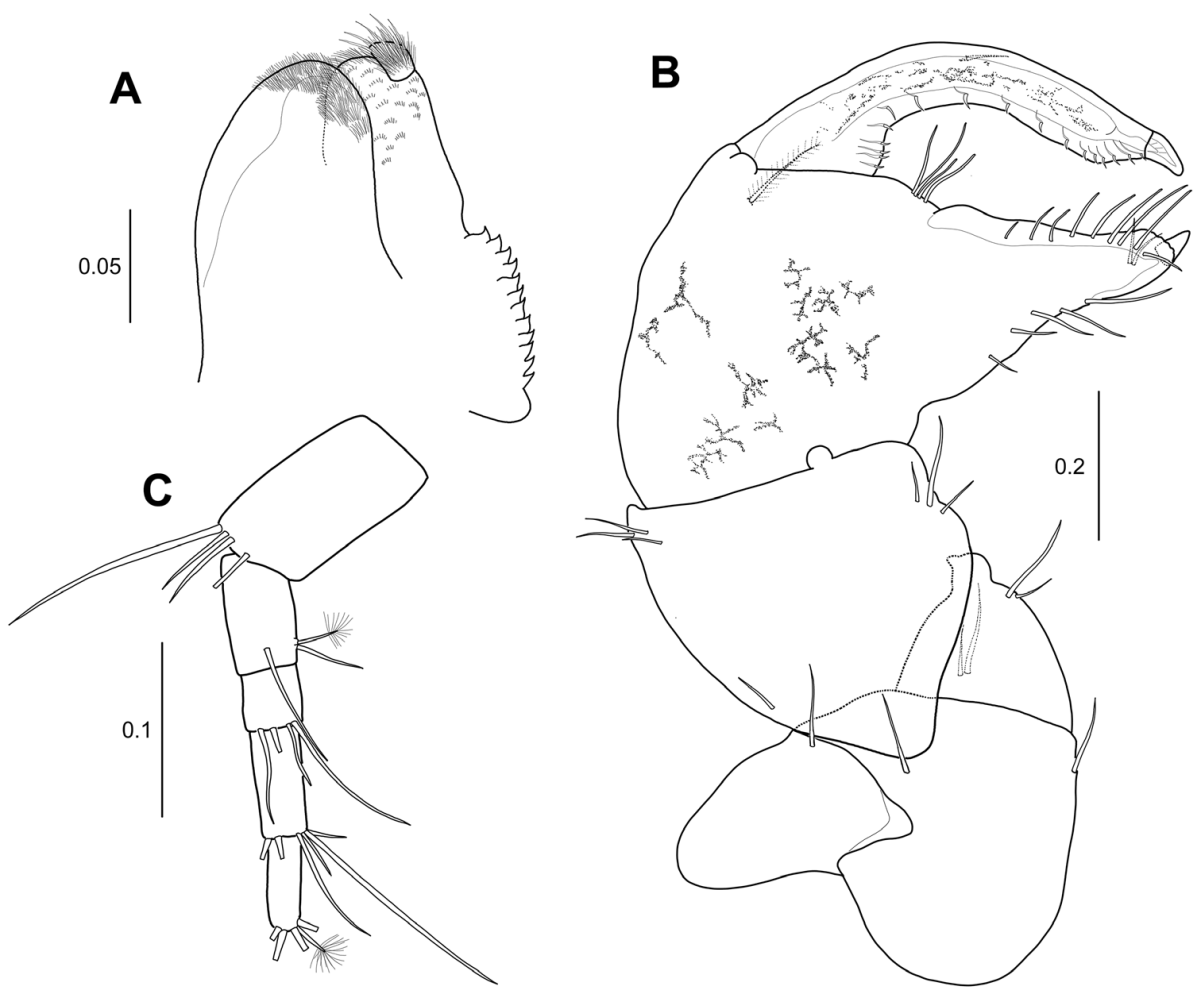
*Hexapleomera ulsana* sp. n., Jeju population, male, **A** labium **B** cheliped **C** uropod. Scale bars in mm.

Appendages similar to those of female except for antennule, antenna, mandibles, cheliped, and uropod:

***Antennule*** (Fig. 12B): 1.1 times as long as cephalothorax. Proportional length of articles 61.3: 21.2: 15.8: 1.7. Article 1 4.2 times as long as wide, with one outer medial broom seta, two outer distal broom setae, three simple setae and two broom setae on subdistal margin, and one inner distal simple seta. Article 2 1.8 times as long as wide, with eight distal broom setae. Article 3 1.9 times as long as wide, with two broom setae on subdistal margin. Article 4 with four aesthetascs and several simple setae.

***Antenna*** (Fig. 12C): 0.9 times as long as antennule. Proportional lengths of articles 9.3: 26.7: 11.8: 28.7: 16.7: 6.8. Article 1 naked. Article 2 with two medial simple setae, one outer distal seta, and proximally microtrichia. Article 3 with one distal simple seta. Article 4 longest, with three distal simple setae. Article 5 with three simple setae and three broom setae. Article 6 with one broom seta and 14 simple setae on distal margin.

***Left mandible*** (Fig. 11D): Two setae of setal row smaller than those of female. *Right mandible* (Fig. 11E): Lacinia mobilis reduced to blunt spine, about twice as long as two pinnate setae of setal row.

***Labium*** (Fig. 12A): Lobe with 12 lateral spines; labial palp 1.6 times as long as wide.

***Cheliped*** (Fig. 12B): Basis 1.1 times as long as wide, with one ventro-subdistal seta and one dorso-subdistal seta. Merus slightly protruded on ventrodistal margin, with two ventromedial setae and two dorsal setae. Carpus equal to basis in length and width, with three ventromedial setae, one dorso-subproximal seta, and three dorsodistal setae. Propodus 1.8 times as long as wide, with one setulose inner seta and four simple setae near insertion of dactylus. Fixed finger with five simple setae along ventral margin and nine simple setae along cutting edge and two inner simple setae. Dactylus with 17 small denticles along cutting edge and one inner medial seta.

***Uropod*** (Fig. 12C): Basal article 1.8 times as long as wide, with four distal setae.

#### Remarks

Two populations of *Hexapleomera ulsana* from the Ulsan and Jeju Island exhibited a morphological divergence in the number of the setae on the maxilliped coxa (3 vs. 2), setae on the maxillule palp (4 vs. 6), setae on the pleopod basal article (absence vs. presence), pattern of pigmentation of cephalothorax, and the number of ventral setae on the pereopods 2–3 propodus (3 vs. 2). On the other hand, specimens of both populations coincided in the number of uropod articles (5), the presence of apophysis on the pereopod 1 coxa, the number of setae on the mandible setal row, and the number of setae on the maxilliped endite. Little morphological variation was found in each population. These results show that the geographical isolation can affect the morphological divergence between two populations of *H. ulsana*.

#### Molecular results

The mtCOI gene sequences between two populations of *H. ulsana* from Ulsan and Jeju Island showed somewhat low molecular divergence (1.1 %). The divergence values are genetically indicative of the same species when compared with other taxa of Tanaididae (Larsen et al. 2014, in table 2), in which different species of *Zeuxo* represented a genetic divergence in the mtCOI gene ranging from 10.7 % to 28.5 %. Therefore, the genetic results may mean that separation at the population level is ongoing. Genetic divergence of the mtCOI between *H. ulsana* and *H. urashima* (Japan) was distinct (32.4 %) revealing the two species to be different. The sequences of mtCOI of two populations of *H. ulsana* were submitted to GenBank under the accession numbers of KY303901–KY303903 (Ulsan) and KY303904–KY303905 (Jeju Island), respectively: KY303901 was extracted from the female paratype (MABIK CR00240699), KY303902 from the female paratype (MABIK CR00240701), KY303903 from the male paratype (MABIK CR00240704) of the Ulsan population; KY303904 from the female paratype (MABIK CR00240707) and KY303905 from the male paratype (MABIK CR00240710) of the Jeju population.

## Discussion

The family Tanaididae Nobili, 1906 consists of five subfamilies, 19 genera, and 87 species (Anderson 2017). The subfamily Pancolinae Sieg, 1980 is divided into two tribes, Anatanaini Sieg, 1980 and Pancolini Sieg, 1980. Pancolini includes five genera, including *Aviatanais* Bamber, 2005, *Hexapleomera* Dudich, 1931, *Monoditanais* Sieg, 1980, *Pancoloides* Sieg, 1980, and *Pancolus* Richardson, 1905. The tribe is defined by the reduction of pleonites 3–5, pleopod 3 being smaller than pleopods 1–2, and sexual dimorphism in the cheliped, antennule, and antenna (Sieg 1980). Until quite recently, several species of *Hexapleomera* have been recorded as *H. robusta* from diverse regions (Anderson 2017, page 648–650), which has been regarded as cosmopolitan species due to a unique ecology related to sea-going turtles as their host. Bamber (2012) re-assessed *Hexapleomera* by examining previous literature and material of free-living benthic specimens from the eastern Mediterranean; he concluded that the genus was comprised by seven species. Currently, the genus contains nine species including new species of this study. To clarify species described in the present study to be new, Table 1 presents a comparison of the morphological characteristics between both sexes of new species from Korea and eight previously described species of *Hexapleomera*.

### Morphological comparison among nine species within the genus *Hexapleomera*

Nine species of *Hexapleomera* were compared based on the combination of morphological characteristics (Table 1). These characters include the presence/absence of coxa of the pereopod 1, the number of uropod article, the number of inner setae on the pleopod 3 basal article, the number of setae on the maxilliped palp, the setae on the basis, coxa, and the endite of the maxilliped, which were suggested diagnostic features of species within the genus by Bamber (2012, in Table 1). Recently, Tanabe et al. (2017) recorded the stable and variable characters of species in *Hexapleomera* based on the morphological and genetic examination of *H. urashima*. As a result, the number of uropodal articles, setae of pleopod 3 basal article, setae of maxilliped coxa, and endite were confirmed to have stability. The number of setae of the mandible setal row and the number of ventral setae on the pereopods 2–3 propodus were newly added as key characters in this study, which showed difference in *H. edgari*, *H. robusta* *sensu* Sieg, *H. satella*, and *H. wombat* but equal in *H. bultidactyla* and *H. moverleyi* (2 and 2) and *H. ulsana* and *H. urashima* (3 and 3). In the case of the mandible, some authors (Larsen and Wilson 1998, Edgar 2008) mentioned that the form and number of setae consisting of the setal row of mandibles varied with growth as well as between individuals within populations. However, those of *H. ulsana* showed difference only in size. The presence of an inner seta on the pleopod 3 basal article was found only in two species from Australia (*H. edgari* and *H. moverleyi*). Two setae of the mandible setal row were present only in *H. robusta* *sensu* Sieg, *H. ulsana*, and *H. urashima*, while the other species contained only one seta on the mandible setal row.

## Supplementary Material

XML Treatment for Hexapleomera ulsana
